# Why medical professionals must learn mathematics and computing?

**DOI:** 10.12669/pjms.40.2(ICON).8952

**Published:** 2024-01

**Authors:** Javeria Aijaz

**Affiliations:** 1 Javeria Aijaz, FCPS, PhD. Section Head, Molecular Biology, Pathology Department Indus Hospital & Health Network, Karachi, Pakistan. Email: javeria.aijaz@tih.org.pk

Biology, mathematics, and computing have been developing independently for years. Many students lacking a particular penchant for mathematics have thus chosen medical and biological sciences to serve this disinclination, if not for any other reason. With the recent blurring of inter-disciplinary boundaries between these fields,^1^ however, insulation of medical and biological sciences from mathematics and computation is rapidly dissipating. The confluence has already had exciting outcomes,^2^ while its unleashed potential is expected to radically transform every aspect of modern medicine.

One area of major impact currently is genetics and genomics, propelled by advances in DNA sequencing technology.^3^ The explosion of information generated by sequencing, and its practical applications, are currently only limited, if so, by matching advances in computational power and tools, though these are also catching up rapidly. Machine learning algorithms, currently developing at breakneck speed, generate information from sequencing data through identifying patterns that are otherwise elusive to the human mind. Sequencing, and the associated machine learning algorithms, have already begun to displace traditional diagnostics.^4^ These not only provide more accurate and useful diagnostic information, but also are much more cost-effective than the resource-intensive, time-consuming traditional pathological methods.

Machine learning algorithms can analyze vast amounts of medical data as well, including patient histories, laboratory results, and imaging scans, to identify patterns and make accurate diagnoses.^5^ In the future, AI’s ability to analyze large datasets and mine valuable insights will enable the development of personalized treatment plans for patients. By considering an individual’s unique genetic makeup, medical history, and other factors, AI algorithms can suggest tailored interventions, medication options, and lifestyle modifications. Nonetheless, at least for the foreseeable future, medical practitioners may continue to play a crucial role in interpreting AI-generated recommendations, ensuring patient understanding, and adapting treatment plans based on a holistic understanding of the patient’s condition.

Another area where biology, mathematics, and computing are intersecting is in the design of targeted drug therapies. Computer simulations can predict how different drugs will interact with various proteins in the body.^6^ Combined with basic knowledge of the pathogenesis of various diseases, rapid screening of potential therapies can be possible, exponentially fast-tracking drug development processes. In the same vein, the study of brain at the molecular, cellular, and systems levels, has enabled new technologies like brain-computer interfaces that can help people with disabilities or injuries. Artificial neural networks, modeled after the structure of the brain can learn and adapt to new situations, allowing them to perform tasks such as image recognition and natural language processing with high accuracy.^7^

The increasing reliance on electronic health records and healthcare informatics necessitates proficiency in computational tools. Medical professionals must navigate and analyze vast amounts of patient data efficiently. Knowledge of computational techniques, such as data mining, machine learning, and artificial intelligence, will equip healthcare professionals to extract valuable insights, improve clinical decision-making, and enhance patient care outcomes^8^. Additionally, medical breakthroughs often rely on interdisciplinary collaborations between mathematicians, statisticians, and medical professionals.

While a complete listing of the ways in which the convergence of biology, mathematics, and computation will transform the practice of medicine is a challenging, if not impossible, task, the fact that progress in the medical field is now led by its confluence with mathematics and computation is incontrovertible. Incorporation of mathematics and computation in biological and medical curricula is hence an unmet need currently.^9^ Urgent action on this front is imperative if our medical professionals and scientists are to keep up pace with modern medicine and research, and before much of the knowledge and skills acquired during their education and training are rendered obsolete.
